# The Impact of Parental Involvement in the Prevention and Management of Obesity in Children: A Systematic Review and Meta-Analysis of Randomized Controlled Trials

**DOI:** 10.3390/children11060739

**Published:** 2024-06-17

**Authors:** Abdulsalam M. Aleid, Noor M. Sabi, Ghaida Saleh Alharbi, Atheer A. Alharthi, Sarah M. Alshuqayfi, Nirmeen S. Alnefiae, Gawaher M. Ismail, Abdulaziz K. Allhybi, Awatif M. Alrasheeday, Bushra Alshammari, Yasmine Alabbasi, Abbas Al Mutair

**Affiliations:** 1 Department of Surgery, Medical College, King Faisal University, Hofuf 31982, Saudi Arabia; 2 Department of Medicine, Medical College, Umm Al-Qura University, Makkah 31982, Saudi Arabia; 3 Unaizah College of Medicine, Qassim University, Unaizah 24221, Saudi Arabia; 4 College of Medicine, Taif University, Taif 51911, Saudi Arabia; 5 College of Medicine, Alexandria University, Alexandria 26571, Egypt; 6 College of Medicine, King Saud bin Abdulaziz University for Health Sciences, Jeddah 21561, Saudi Arabia; 7 Nursing Administration Department, College of Nursing, University of Hail, Hail 21424, Saudi Arabia; 8 Medical Surgical Nursing Department, College of Nursing, University of Hail, Hail 2440, Saudi Arabia; 9 Department of Maternity and Pediatric Nursing, College of Nursing, Princess Nourah bint Abdulrahman University, Riyadh 11671, Saudi Arabia; 10 Department of Medical-Surgical Nursing, Princess Nourah bint Abdulrahman University, Riyadh 11671, Saudi Arabia

**Keywords:** caregivers, prevention, pediatrics, BMI z-score, exercise levels, screen time, dietary self-efficacy, percentage body fat

## Abstract

Background: Obesity in children is a critical public health issue in developed countries and developing countries. The establishment of health-related behaviors in childhood, significantly influenced by parental involvement, underscores the need for effective intervention measures. Aim: This original research is a systematic review and meta-analysis that aimed to investigate the impact of parental involvement on the prevention and management of childhood obesity, focusing on outcomes such as BMI z-score, exercise levels, screen time, dietary self-efficacy, and percentage body fat. Methods: Adhering to the PRISMA guidelines, we conducted a systematic review and meta-analysis of 12 randomized controlled trials (RCTs) identified through comprehensive searches of PubMed, Scopus, Web of Science, and the Cochrane Library, including RCTs involving children aged 2–18 years with parental or caregiver participation, reporting on the specified outcomes. Data analysis was performed using RevMan 5.3, employing a random effects model. Results: A total of 5573 participants were included. The meta-analysis revealed a significant reduction in BMI z-score (MD = −0.06, 95% CI: −0.09 to −0.02, *p* = 0.005, I^2^ = 58%), a non-significant increase in exercise levels (SMD = 0.26, 95% CI: −0.01 to 0.52, *p* = 0.05, I^2^ = 52%), and a significant reduction in screen time (MD = −0.36 h per day, 95% CI: −0.61 to −0.11, *p* = 0.005, I^2^ = 0%). Dietary self-efficacy also improved significantly (MD = 0.59, 95% CI: 0.12 to 1.05, *p* = 0.01, I^2^ = 0%). However, changes in percentage body fat did not reach statistical significance (MD = −1.19%, 95% CI: −2.8% to 0.41%, *p* = 0.15, I^2^ = 0%). Conclusion: Parental involvement in childhood obesity interventions significantly impacts BMI z-score, exercise levels, screen time, and dietary self-efficacy but not percentage body fat. These findings highlight the importance of engaging parents in obesity prevention and management strategies.

## 1. Introduction

Approximately 23% of children in developed countries and 13% in developing countries are estimated to be impacted by childhood obesity, which remains a significant public health concern worldwide. The critical need for efficient preventative and intervention measures is highlighted by this concerning trend [1]. Childhood is a crucial time for the development of health-related behaviors that greatly increase the risk of obesity, such as eating habits, levels of physical activity, and screen time [2]. In this situation, parents’ influence as the main caretakers and role models is crucial in forming these habits [3,4,5].

The significance of parental engagement in therapies meant to fight obesity in children has been emphasized in recent work. The efficacy of parent-involved treatments has been highlighted in systematic reviews and meta-analyses, which show that these approaches have a greater influence on children’s body mass index (BMI) and health behaviors than non-family-involved therapies [4,5,6,7]. Remarkably, a 2012 meta-analysis revealed that parental participation programs resulted in higher BMI reductions for children aged 2 to 19 years compared to those without such involvement [4]. Despite these realizations, there are still gaps in our knowledge, especially when it comes to the age at which interventions work best and the outcomes other than BMI that parental involvement may affect.

Furthermore, the effectiveness of parent-focused therapies in the preschool age range (0–6 years) has been less evident, with conflicting findings on long-term consequences [3,8]. This indicates that a more thorough investigation is required to determine the precise elements that influence the effectiveness of these treatments, such as the age at which interventions are started and the children’s baseline BMI.

This original research is systematic review and meta-analysis that aimed to investigate the role of family involvement in preventing obesity in children and the impacts of parents’ participations in managing plans on the outcomes. We hypothesized that family involvement positively affects screen time duration, activity levels, BMI z-score, dietary self-efficacy, and % body fat outcomes in children with obesity.

## 2. Materials and Methods

Following the Preferred Reporting Items for Systematic Reviews and Meta-Analysis (PRISMA) guidelines, we conducted a systematic review and meta-analysis. This comprehensive review encompassed all clinical randomized trials that assessed the effectiveness and outcomes of parents’ involvement and role in preventing obesity in children. This rigorous methodology allowed us to systematically gather and analyze the available evidence, providing valuable insights into the potential benefits of these interventions in childhood obesity outcomes [9].

A comprehensive literature search was conducted using PubMed, Scopus, Web of Science, and Cochrane library databases. The search focused on RCTs published in English, comparing parents’ involvement in managing and preventing childhood obesity versus control groups. Keywords related to childhood obesity, parents, prevention, and family role were combined in various ways to maximize the search scope.

Initial search results were screened based on titles and abstracts for relevance to the research question. Studies that appeared to meet the inclusion criteria had their full texts reviewed. This process resulted in the final inclusion of 12 RCTs in the meta-analysis.

### 2.1. Study Selection

**Figure 1:**
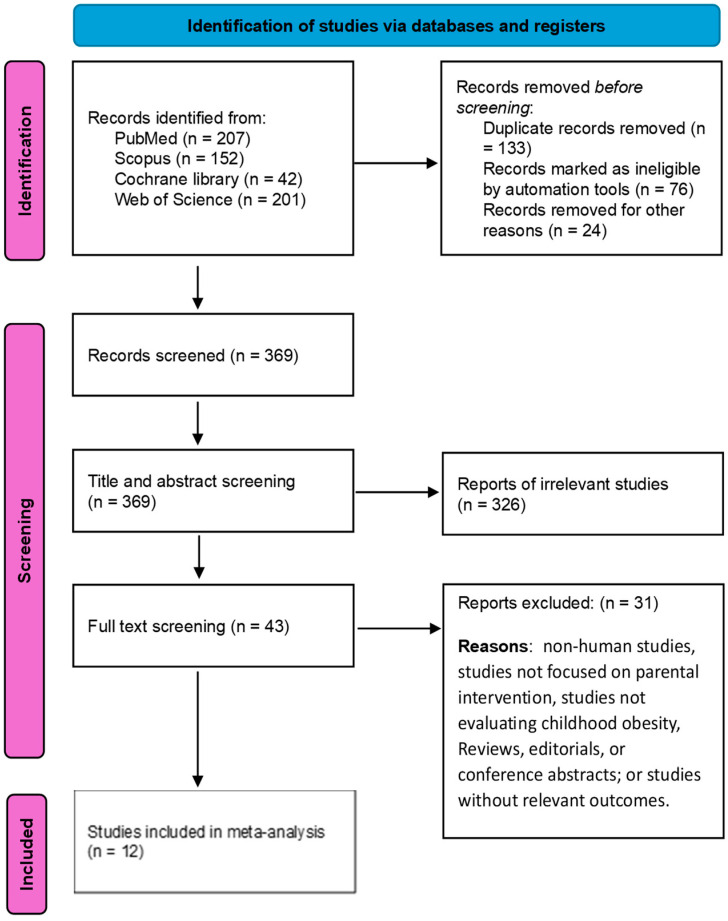
PRISMA flowchart of included studies.

This process was carried out by two authors. Both authors thoroughly assessed the full papers, and any discrepancies or disagreements that arose during the evaluation were resolved through a consensus reached between the two authors. We report our selection process in the PRISMA diagram flowchart (Figure 1).

### 2.2. Inclusion and Exclusion Criteria

**Inclusion Criteria:**

- Study Design: Only RCTs were included to ensure a high level of evidence in evaluating the effects of parental involvement on childhood obesity outcomes.
- Participants: Studies involving children aged 2–18 years, with a clinical diagnosis of obesity, were included. Parental or caregiver involvement in the intervention was required.
- Interventions: Interventions that actively involved parents or caregivers in strategies aimed at preventing childhood obesity. This could include dietary education, physical activity promotion, screen time management, and other behavioral interventions.
- Outcomes: Studies must report on at least one of the following outcomes: BMI z-score, exercise levels, screen time, dietary self-efficacy, and percentage body fat.

**Exclusion Criteria:**

- Study Design: Non-randomized studies, observational studies, case reports, reviews, and meta-analyses were excluded to maintain the methodological quality of the review.
- Participants: Studies focusing exclusively on adults, or interventions not involving parental or caregiver participation, were excluded.
- Interventions: Studies focusing on pharmacological, surgical, or other clinical treatments for obesity without a behavioral intervention component involving parents were excluded.
- Outcomes: Studies not reporting any of the predefined outcomes or lacking precise outcome measures were excluded.

### 2.3. Data Extraction

We extracted all relevant data from the selected studies and organized them into a dedicated spreadsheet. This spreadsheet captured essential information such as the study design; country; follow-up; total number of intervention and control groups; the age of both intervention and control groups reported as mean and SD, BMI, sex, and main findings. Data extraction was carried out by two independent reviewers. Discrepancies were resolved through discussion or third-party judgment.

### 2.4. Quality Assessment Using ROB2 Tool

The ROB2 tool was used to assess the risk of bias in RCTs and non-randomized studies. This tool evaluates bias arising from the randomization process, deviations from intended interventions, missing outcome data, measurement of the outcome, and selection of the reported result [10]. We used Robvis online software to generate traffic light plot for quality assessment at 26 February 2024 and the software URL is: https://mcguinlu.shinyapps.io/robvis/.

The researchers selected 12 relevant research articles for a quality assessment. Two of the researchers independently reviewed each research article. If discrepancies were found in the evaluation, they were resolved through a discussion and consensus. Each article was assigned a high, low, or with some concerns of bias for each domain. The final evaluation provided a complete summary of the quality methodologies of the included articles.

### 2.5. Outcomes Measured

Primary outcomes of interest were BMI z-score, exercise, and screen time. Secondary outcomes included dietary self-efficacy and % body fat.

### 2.6. Statistical Analysis and Heterogeneity

We used RevMan 5.3 software to perform the analysis. Changes in continuous variables (BMI z-score, exercise, screen time, dietary self-efficacy, and % body fat) were pooled as a mean difference (MD) and standardized mean difference (SMD) for exercise due to different scales that were used to assess this outcome. The random effects model was adopted.

To determine heterogeneity, visual assessment of the forest plots was used, while the chi-square (χ^2^) and I^2^ tests were used to measure it. Heterogeneity was assessed using the χ^2^ test, and if it was found, quantification was performed using the I^2^ test. To interpret the I^2^ test, the meta-analysis guidelines of the Cochrane Handbook were followed (75–100% = significant heterogeneity, 50–90% = may represent substantial heterogeneity, 30–60% = may represent moderate heterogeneity, and 0–40% = may not be significant) [11].

## 3. Results

### 3.1. Literature Search

Figure 1 displays a flow chart of papers selected and included following PRISMA standards [9]. An electronic search of databases identified 602 records; 369 were included in the title and abstract screening, and the remaining 233 were duplicates; 326 were excluded as they did not meet our inclusion criteria. We conducted the full-text screening on the eligible 43 studies. By full-text screening, 12 studies with 5573 patients met our inclusion criteria and were included in the present analysis (Figure 1).

### 3.2. Characteristics of the Included Studies

The 12 included studies encompassed a total of 5573 patients. These studies originated from various global regions offering a broad representation of the target population. A summary of characteristics is found in Table 1.

### 3.3. Quality Assessment of Included Studies

**Figure 2:**
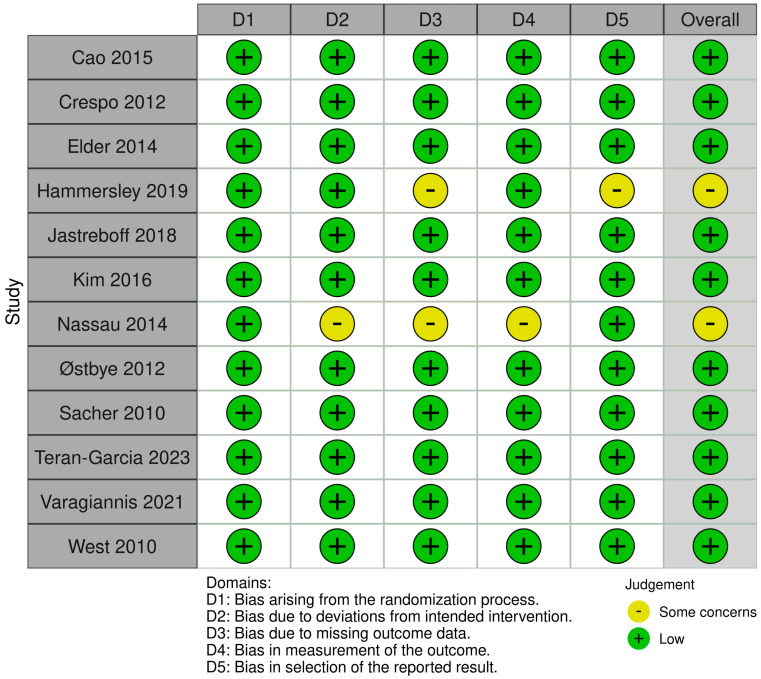
Quality assessment of included studies using the ROB2 tool [12,13,14,15,16,17,18,19,20,21,22,23].

The methodological quality of each study was rigorously assessed using the Cochrane Risk of Bias Tool for Randomized Trials (ROB 2) [10]. Among the included studies, 10 studies were rated as having a low risk of bias across all domains, indicating a strong methodological approach. However, two studies identified some concerns as they received an unclear rating in at least one domain (Figure 2).

These results show the importance of considering the methodological quality of individual studies when interpreting the synthesized evidence.

### 3.4. Data Analysis

#### 3.4.1. BMI Z-Score

**Figure 3:**
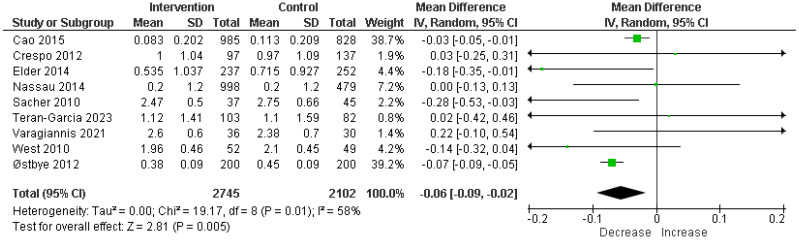
Analysis of mean difference BMI z-score in intervention vs. control [12,13,14,18,19,20,21,22,23].

The meta-analysis of 9 RCTs including a total of 4847 participants showed a significant reduction in BMI z-score. The pooled MD was −0.06, with a 95% confidence interval (CI) of −0.09 to −0.02, indicating a statistically significant effect (*p* = 0.005) of parental involvement on reducing BMI z-score in children; the heterogeneity was moderate (I^2^ = 58%) (Figure 3).

#### 3.4.2. Exercise

**Figure 4:**
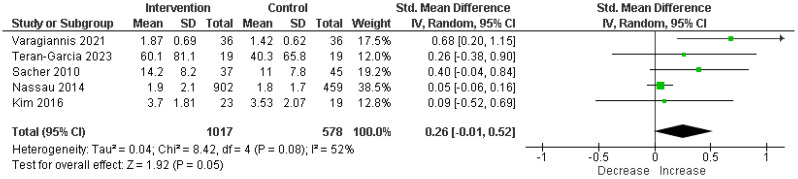
Analysis of standardized mean difference exercise in intervention vs. control [17,18,20,21,23].

For exercise levels, the analysis of five RCTs comprising 1595 participants indicated no significant increase, employing an SMD to account for varying scales across studies. The SMD was 0.26 with a 95% CI of −0.01 to 0.52, demonstrating a statistically significant enhancement in physical activity levels (*p* = 0.05) attributable to parental involvement; the heterogeneity was moderate (I^2^ = 52%) (Figure 4).

#### 3.4.3. Screen Time

**Figure 5:**
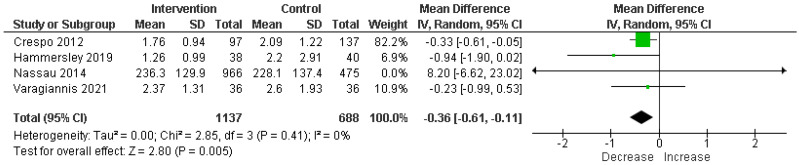
Analysis of mean difference screen time in intervention vs. control [13,15,18,21].

A significant reduction in screen time was observed across four RCTs involving 1825 participants. MD was −0.36 h per day, with a 95% CI of −0.61 to −0.11, showcasing a statistically significant decrease (*p* = 0.005) in screen time due to the intervention; there was no heterogeneity (I^2^ = 0%) (Figure 5).

##### Dietary Self-Efficacy

**Figure 6:**
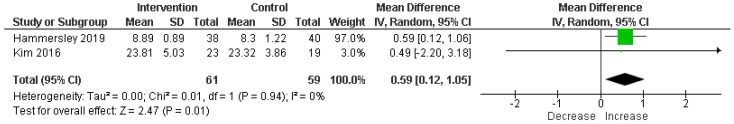
Analysis of mean difference dietary self-efficacy in intervention vs. control [15,17].

Increased dietary self-efficacy was noted among children participating in two RCTs with 120 participants in total. MD was 0.59 on a validated dietary self-efficacy scale, with a 95% CI of 0.12 to 1.05, indicating a statistically significant improvement (*p* = 0.01) in dietary self-efficacy; there was no heterogeneity (I^2^ = 0%) (Figure 6).

##### Percentage Body Fat

**Figure 7:**
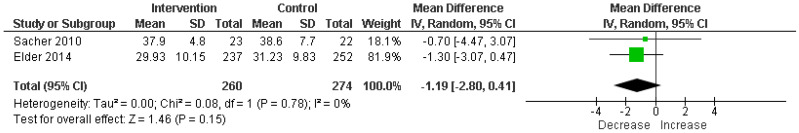
Analysis of mean difference % body fat in intervention vs. control [14,20].

In contrast, the analysis of percentage body fat did not yield statistically significant results. Among two RCTs including 534 participants, MD was −1.19%, with a 95% CI of −2.8% to 0.41%, failing to reach statistical significance (*p* = 0.15); there was no heterogeneity (I^2^ = 0%) (Figure 7).

## 4. Discussion

This original research, a systematic review and meta-analysis aimed to investigate the role of family involvement in preventing obesity in children and the impacts of parents’ participations in managing plans on the outcomes. With 5573 individuals from 12 randomized controlled trials, this systematic review and meta-analysis provide strong proof of the important role parents play in managing and preventing childhood obesity. Our study reveals significant enhancement in several domains, including decreases in children’s BMI z-score and screen time, as well as major increases in their levels of activity and nutritional self-efficacy. These results support the crucial role that parental involvement plays in influencing children’s health-related behaviors and outcomes. Still, the lack of a statistically significant effect on % body fat adds a level of complexity that calls for more research.

Our results demonstrated a significant reduction in BMI z-score, with a pooled MD of −0.06 (95% CI: −0.09 to −0.02, *p* = 0.005), against a backdrop of moderate heterogeneity (I^2^ = 58%). This finding starkly contrasts with the findings of Hammersley et al. [7], where no significant differences were reported for BMI, BMI z-score, or percentage body fat across most studies, except for a transient decrease in percentage body fat at 6 months in one study (−1.12 ± 0.47 SE, *p* < 0.05), which was not sustained at the 2-year mark. Furthermore, our analysis revealed a significant increase in exercise levels (SMD = 0.26, 95% CI: −0.01 to 0.52, *p* = 0.05) with moderate heterogeneity (I^2^ = 52%). This is in comparison to the findings of Hammersley et al. [7], where only one out of six studies assessing physical activity found a significant difference, highlighting the enhanced efficacy of interventions incorporating parental involvement in promoting physical activity among children. Screen time outcomes from our study also showed a significant reduction (MD = −0.36 h per day, 95% CI: −0.61 to −0.11, *p* = 0.005) with no observed heterogeneity (I^2^ = 0%), diverging from the findings of Hammersley et al., where no significant difference in screen time was reported, underscoring the potential of parental involvement to address this risk factor more effectively [7].

Norman et al. emphasizes the complex long-term effects of school-based treatments with parental support, showing that the long-term maintenance of sustained behavioral changes is difficult, especially regarding eating patterns and BMI reduction. This demonstrates the intricate relationships between the long-lasting effects of lifestyle modifications and the effectiveness of the original intervention, implying that further integrated and ongoing measures could be needed for long-term success. Significantly, the varying results depending on gender and the amount of the intervention suggest that customized strategies that consider individual and family circumstances are required, emphasizing the vital role that deeper, more regular parent engagement plays [24].

Building on this, Kim et al.’s study shows how parental engagement treatments may improve children’s nutritional self-efficacy and child–parent interactions. This study demonstrates how parent-only treatments can result in notable enhancements to child–parent interaction and children’s self-assurance in making food decisions. The brief intervention’s length, however, highlights the need for longer-term, ongoing engagement tactics to significantly alter BMI [17].

The NOURISH program pilot research by Bean et al. offers more proof of the beneficial impacts of parent-focused interventions on dietary practices. The study indicates that minimal intervention may be just as successful as organized programs in eliciting short-term dietary changes, even though both the intervention and control groups reported improvements in their diets. This research suggests that even a little amount of parental participation may have a significant impact on how well-behaved children grow up and provides opportunities to investigate scalable, reasonably priced methods of managing childhood obesity [25].

To better understand the varying outcomes of parental involvement in childhood obesity interventions, it is crucial to consider several influential factors. Firstly, parental motivation and engagement are critical, as the level of enthusiasm and commitment parents bring to an intervention can significantly affect its success. Variations in this engagement can lead to different outcomes, even among similar demographics. Secondly, socioeconomic factors play a substantial role; families from different economic backgrounds may have disparate access to resources such as nutritious foods and safe environments for physical activity, influencing the efficacy of any intervention [26]. Additionally, cultural influences can affect dietary preferences and lifestyle habits, necessitating culturally tailored interventions to ensure relevance and effectiveness. Age and developmental factors also demand consideration; interventions may need to be adjusted based on the child’s age to address specific developmental needs and capabilities effectively [27].

When taken as a whole, these findings highlight a crucial issue: parents’ critical involvement in managing and preventing childhood obesity. Parental participation has improved physical activity levels, nutritional self-efficacy, and general child–parent interactions, but there are still difficulties in sustaining long-term behavior change and attaining a meaningful BMI decrease. This emphasizes how important it is for treatments to involve parents in addition to being durable, customized to meet the requirements of each family, and incorporated into daily routines for maximum efficacy.

## 5. Limitation

The low number of included studies, as the research focuses mostly on short-term follow-up data, make it impossible to evaluate how sustainable the reported effects would be over the long run. The included studies show considerable heterogeneity in intervention design, duration, and intensity, complicating the ability to generalize findings across different contexts. Additionally, variability in the measurement tools used to assess key outcomes such as dietary intake, physical activity, and screen time introduces challenges in comparing results across studies.

## 6. Conclusions

Our original research, a systematic review and meta-analysis underscore the significant role of parental involvement in the prevention and management of obesity in children, highlighting its positive impact on children’s BMI z-score, exercise levels, dietary self-efficacy, and screen time. These findings reinforce the importance of engaging parents as key stakeholders in obesity intervention strategies, suggesting that their active participation can influence health-related behaviors and outcomes in children. However, the absence of long-term follow-up data and the heterogeneity of intervention designs and measurement tools across studies call for a cautious interpretation of these results. Moreover, the potential for publication bias and the limited generalizability of the findings to diverse socio-demographic contexts suggest the need for further research. Future studies should aim to address these limitations by incorporating a long-term follow-up; standardizing outcome measures; measuring the association between demographics, parental education level, urban vs. rural backgrounds, wealth and outcomes; and ensuring broad representation of populations to fully understand the dynamics of parental involvement in children with obesity interventions.

## Figures and Tables

**Table 1:** Baseline characteristics of included studies.

Study ID	Study Design	Country	Total Sample Size		Age (Mean, SD)		Sex (Male N)		BMI (Mean, SD)		Follow Up	Main Findings
			Intervention	Control	Intervention	Control	Intervention	Control	Intervention	Control		
Cao 2015 [12]	RCT	China	965	889	7.01 0.44	6.81 0.24	529	468	NR		3 years	♦The family-individual-school–based comprehensive intervention model is effective for controlling childhood obesity and overweight.
Crespo 2012 [13]	RCT	USA	198	227	33 ± 6		5		29.7 ± 6.7		2 years	♦A promotor-based behavioral intervention was efficacious at changing parental factors and child obesity-related health behaviors.
Elder 2014 [14]	RCT	USA	271	267	NR		NR		16.96 2.63	17.42 3.18	2 years	♦Favorable implementation fidelity and high retention rates support the feasibility of this intervention in a large metropolitan area;
Hammersley 2019 [15]	RCT	Australia	42	44	3.36 (0.80)	3.55 (1.02)	24 (57)	19 (43)	17.28 (1.44)	16.72 (0.92)	6 months	♦parent-focused eHealth childhood obesity prevention program can provide support to improve dietary-related practices and self-efficacy but was not successful in reducing BMI.
Jastreboff 2018 [16]	RCT	USA	20	22	30.2 (6.3)	31.6 (6.9)	0	1 (5%)	NR		8 weeks	♦mindfulness-based parent stress intervention to decrease childhood obesity risk is feasible,
Kim 2016 [17]	RCT	Korea	23	19	9.70 ± 1.49	9.79 ± 1.62	16	8	24.26 ± 2.74	23.97 ± 2.34	1 year	♦The results support the effectiveness of the parent involvement intervention in promoting child-parent relationship and dietary self-efficacy of children.
Nassau 2014 [18]	RCT	Netherlands	998	479	NR		NR		19.5 3.4	19.6 3.3	20 months	♦DOiT-implementation program had some beneficial effects on specific obesity-related behaviors in subgroups.
Østbye 2012 [19]	RCT	USA	200	200	3.06 (1.0)		(113)	(110)	NR		1 year	♦There were no group differences in the primary outcomes, but differences were observed in the parenting and maternal outcomes and there were trends toward improvement in the preschoolers’ diets.
Sacher 2010 [20]	RCT	UK	60	56	10.3 (1.3)	10.2 (1.3)	22	31	27.2 (3.7)	27.1 (4.9)	6 months	♦High-attendance rates suggest that families found this intensive community-based intervention acceptable.
Teran-Garcia 2023 [20]	RCT	USA	239	187	9.98 ± 2.71	10.36 ± 3.07	(103)	(86)	NR		6 months	♦The Abriendo Caminos intervention effectively prevented unhealthy weight gain in Hispanic children in the short-term,
Varagiannis 2021 [21]	RCT	Greece	36	30	10.0 (9.0, 11.8)	10.0 (9.0, 11.0)	15	14	25.5 (23.8, 27.8)	26.4 (23.5, 28.6)	NR	♦personalized family-based interventions are recommended to successfully improve children’s lifestyle and body weight status.
West 2010 [22]	RCT	Australia	52	49	8.58 1.69	8.50 1.65	16	17	NR		1 year	♦The results support the efficacy of Group Lifestyle Triple P and suggest that parenting influences treatment outcomes.

## Data Availability

All underlying data have been presented in this article.
